# Lyssaviruses and the Fatal Encephalitic Disease Rabies

**DOI:** 10.3389/fimmu.2021.786953

**Published:** 2021-12-02

**Authors:** Terence Peter Scott, Louis Hendrik Nel

**Affiliations:** ^1^ Global Alliance for Rabies Control, Manhattan, KS, United States; ^2^ Department of Biochemistry, Genetics and Microbiology, Faculty of Natural and Agricultural Sciences, University of Pretoria, Pretoria, South Africa

**Keywords:** Rabies, lyssavirus, encephalitis, zoonosis, immune evasion, pathophysiology

## Abstract

Lyssaviruses cause the disease rabies, which is a fatal encephalitic disease resulting in approximately 59,000 human deaths annually. The prototype species, rabies lyssavirus, is the most prevalent of all lyssaviruses and poses the greatest public health threat. In Africa, six confirmed and one putative species of lyssavirus have been identified. Rabies lyssavirus remains endemic throughout mainland Africa, where the domestic dog is the primary reservoir – resulting in the highest per capita death rate from rabies globally. Rabies is typically transmitted through the injection of virus-laden saliva through a bite or scratch from an infected animal. Due to the inhibition of specific immune responses by multifunctional viral proteins, the virus usually replicates at low levels in the muscle tissue and subsequently enters the peripheral nervous system at the neuromuscular junction. Pathogenic rabies lyssavirus strains inhibit innate immune signaling and induce cellular apoptosis as the virus progresses to the central nervous system and brain using viral protein facilitated retrograde axonal transport. Rabies manifests in two different forms - the encephalitic and the paralytic form - with differing clinical manifestations and survival times. Disease symptoms are thought to be due mitochondrial dysfunction, rather than neuronal apoptosis. While much is known about rabies, there remain many gaps in knowledge about the neuropathology of the disease. It should be emphasized however, that rabies is vaccine preventable and dog-mediated human rabies has been eliminated in various countries. The global elimination of dog-mediated human rabies in the foreseeable future is therefore an entirely feasible goal.

## Introduction

Lyssaviruses are responsible for rabies, which is arguably the deadliest encephalitic disease known. The prototype, rabies lyssavirus (RABV), is thought to be able to infect all terrestrial mammals. Transmission is through virus-laden saliva, typically through the bite of an infected animal, but sometimes through other means such as scratches and in rare occasions, organ transplants and other means (1, 2). The genus *Lyssavirus* (family *Rhabdoviridae*) is presently composed of 17 viral species and one putative (3). All lyssaviruses are bullet-shaped particles containing negative sense RNA genomes of approximately 11 000 nucleotides in length. The genome encodes 5 structural proteins, namely the nucleoprotein, phosphoprotein, matrix protein, glycoprotein, and the polymerase (5’-N-P-M-G-L-3’) with a 5’ – 3’ transcriptional bias (4, 5). The N protein encapsidates the viral RNA, and together with the P and L proteins, forms the ribonucleoprotein (RNP) complex, which can initiate viral transcription and replication (6). The M protein condenses the RNP into the characteristic bullet-shape and recruits the RNP to the cellular membrane during replication. The M protein is also essential for the budding of the enveloped virus from the cell and specifically interacts with the G protein – also known as the transmembrane spike protein, which is the primary antigenic determinant (7, 8).

RABV is not only the type species of the genus, but by far poses the most significant public health threat among all the lyssaviruses. The domestic dog is the primary reservoir for RABV in dog-rabies endemic countries, but several other terrestrial mammalian species can maintain transmission – most notably carnivores such as raccoons, skunks, foxes, and jackals.

## The Global Burden of Dog Rabies

Globally, an estimated 59,000 people die from dog-mediated rabies every year, of which approximately 40% are children under the age of 15 years (9). Rabies affects the poorest and most underserved communities, with the burden being greatest in developing countries of Africa and Asia (10). However, the disease is seriously underreported for a variety of reasons and remains among the most significant diseases of neglect in the world (11).

By continent, Africa has the second highest burden of rabies, with an estimated 23,500 deaths annually, and has the highest per capita death rate (9). RABV is endemic throughout mainland Africa, with only a handful of island nations having never detected rabies in domestic or wildlife species (e.g., La Réunion, Mayotte, Mauritius) (12).

Of the seventeen recognized lyssavirus species, six confirmed and one putative species have been identified in Africa, namely, RABV, Duvenhage virus (DUVV), Lagos bat lyssavirus (LBV), Mokola lyssavirus (MOKV), Ikoma lyssavirus (IKOV), Shimoni Bat Lyssavirus (SHIBV) and the putative Matlo lyssavirus. Of these, only DUVV (n=3), MOKV (n=2) and RABV have been associated with human fatalities (13). While RABV is only associated with non-volant terrestrial mammals in Africa, DUVV and LBV are both associated with bat reservoirs, while IKOV and MOKV have yet unidentified reservoirs (14, 15).

## Pathophysiology

### Viral Entry, Spread and Proliferation

**Figure 1:**
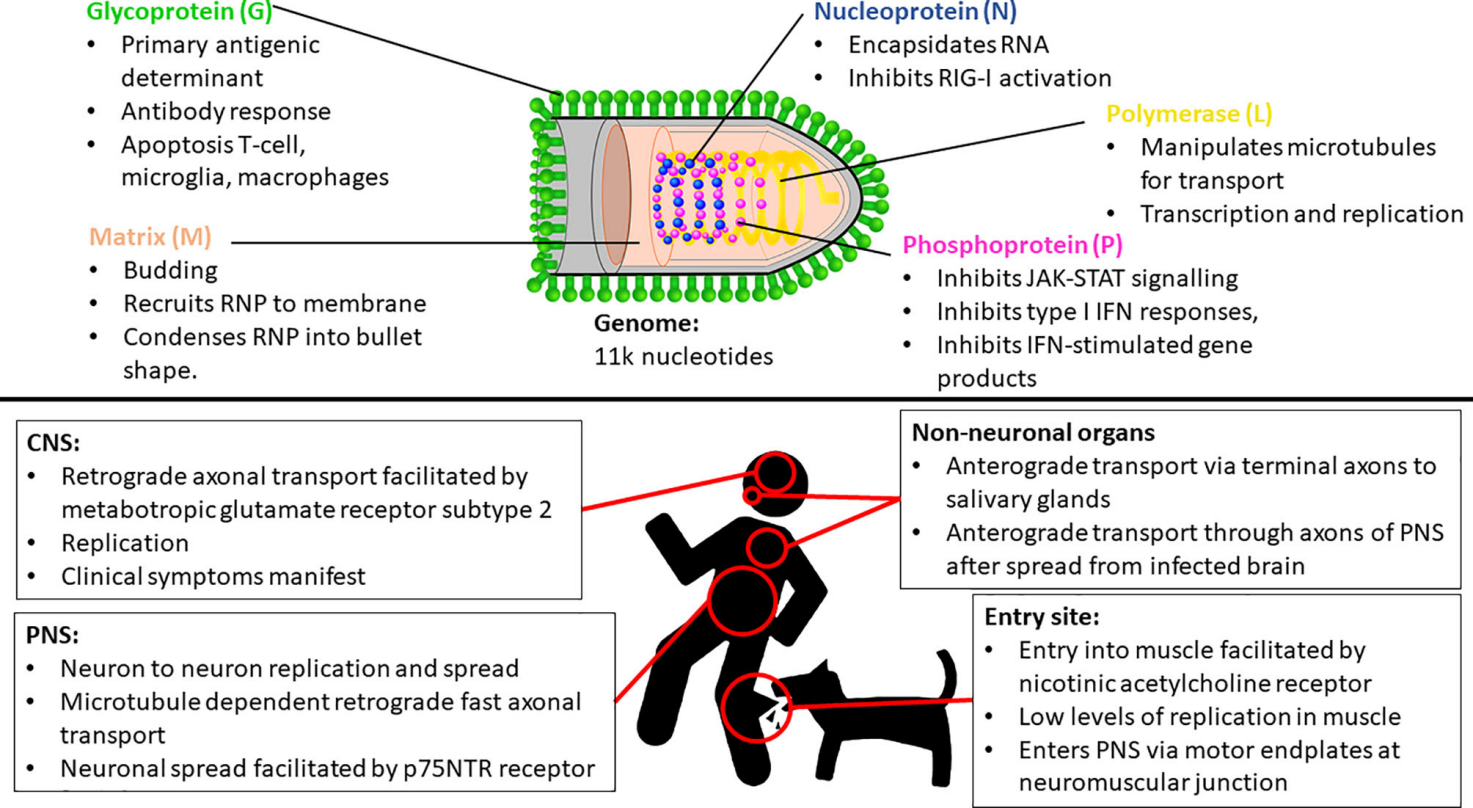
Key insights of Rabies lyssavirus (RABV) entry, spread and proliferation, and some important functionalities of each RABV protein. IFN, Interferon; CNS, Central nervous system; PNS, Peripheral nervous system; RNP, Ribonucleoprotein complex.

The most common method of viral entry is through the injection of virus-containing saliva into the muscle tissue or other peripheral tissue through the bite of an infected animal (**Figure 1**). After inoculation, RABV typically infects muscle cells — thought to be facilitated through the nicotinic acetylcholine receptor — and replicates therein at a low rate (16). The virus remains localized to the inoculation site for variable periods — which may contribute to the variable incubation period characteristic of rabies (17). In contrast, in the case of higher titers of inoculum, RABV can infect motor endplates without the need for the initial replication in the muscle (18). RABV gains entry into the peripheral nervous system (PNS) *via* motor endplates at the neuromuscular junction, but the exact means of virus internalization remains poorly understood.

RABV travels through the PNS towards the CNS *via* microtubule dependent retrograde fast axonal transport (19, 20). The virus travels from neuron to neuron, replicates, and continues its progression towards the CNS and the brain (21). This neuronal spread is facilitated by the p75NTR receptor, which is non-essential for infection, but facilitates directed and more rapid transport of RABV to the CNS (22). The L protein manipulates microtubules for improved transport efficiency (23), while the M protein facilitates the depolymerization of microtubules resulting in improved viral transcription and replication efficiency (24) (**Figure 1**). While retrograde transport occurs at an approximate rate of 50 – 100mm per day in humans [with species-dependent variation (20, 25)], evidence also suggests that RABV undergoes active, G protein-dependent anterograde transport in peripheral neurons - such as Dorsal Route Ganglion (DRG) neurons — at a rate three times faster than that of retrograde transport (25). However, the significance of this anterograde transport mechanism is unclear, but recent evidence signifies its importance in the spread of RABV through the PNS (including to non-neuronal organs) after centrifugal spread from the CNS (26), contrasting previous evidence that suggested that RABV spreads by both axonal and trans-synaptic transport exclusively in the retrograde direction (21, 27). Once in the CNS, RABV continues to spread *via* retrograde axonal transport thought to be facilitated by metabotropic glutamate receptor subtype 2, which is a cellular entry receptor that is abundant throughout the central nervous system (CNS) (28). The virus reaches the brainstem and subsequently the brain, where it proliferates and clinical symptoms manifest. It spreads to the salivary glands along terminal axons *via* anterograde transport (29) where it continues to proliferate and is subsequently shed in the saliva for transmission to another host. RABV can spread to peripheral, non-neuronal organs anterograde transport, and can be detected in these sites after the onset of clinical symptoms (21, 26).

### Symptoms, Disease Progression, Prevention, and Treatment

Rabies presents with a wide variety of clinical manifestations that vary depending on multiple factors, many of which remain unknown. However, the species of lyssavirus or the strain of RABV influences the presentation of differing clinical symptoms. For example, bat RABV infections more commonly present with tremors and involuntary twitching/jerking (myoclonus), while dog strains more frequently present with classical hydrophobia and aerophobia (30). Moreover, the presentation of symptoms localized to the wound were more common in bat rabies exposures than in dog-rabies exposures (30). Two forms of rabies can manifest, namely encephalitic (furious or classical) and paralytic (dumb) rabies. The encephalitic form of rabies is more common and presents in approximately 80% of patients, of which between 50 – 80% present with the classic symptoms such as hydrophobia and aerophobia – symptoms that are unique to rabies (31, 32). However, the remaining symptoms are common to many encephalitic diseases, especially in African countries where diseases such as cerebral malaria are endemic and can result in misdiagnosis of rabies (33). Encephalitic rabies typically progresses to severe flaccid paralysis, coma and death caused by multiple organ failure, in contrast to paralytic rabies which manifests with prominent muscle weakness early in the course of illness (31). While there remains a gap in the understanding of the causes for the manifestation of these two different forms of rabies, it is known that the anatomical site of the exposure is unrelated (34). Initially rabies symptoms were thought to be caused by large-scale neuronal cell death, but neuronal apoptosis is only stimulated during infection with low pathogenicity strains (35, 36). Rather, symptoms are thought to be due to neuronal cell dysfunction (35, 37–41), partly induced by the increased production of Nitric Oxide (NO) *via* inducible nitric oxide synthase (iNOS) in neurons and macrophages (42–44). Elevated levels of NO produced by iNOS leads to mitochondrial dysfunction and as a result, axonal swelling (44, 45) — a pathology that is associated with the onset of symptoms (41, 46), and hypothetically explains the development of encephalitic symptoms (47). Another mechanism behind neurological dysfunction and the onset of neurological symptoms has been demonstrated to be reliant upon a host-derived mechanism that results in the loss of axons and dendrites as a means to prevent the spread of the virus (48).

The survival time for patients manifesting paralytic rabies is approximately 41% longer than that of patients with encephalitic rabies (30, 49), yet the incubation periods for both forms remain similar – ranging from 2 weeks to several months. For most cases, the incubation period is 2 – 3 months in humans, but some exceptional cases have been documented with an incubation period of more than a year and even up to 8 years (50, 51). There is no known accepted treatment for rabies after the onset of clinical symptoms. Palliative care is recommended for rabies patients, which is aimed to reduce suffering and may temporarily prolong survival time, but in all but the most exceptional circumstances, the victim succumbs to the disease (32, 50). However, effective pre- and post-exposure prophylaxis exists for those viruses that fall within lyssavirus phylogroup 1 [RABV, European bat lyssavirus-1 and -2, Bokeloh bat lyssavirus, DUVV, Australian bat lyssavirus, Aravan lyssavirus, Khujand lyssavirus, Irkut lyssavirus, Taiwan bat lyssavirus, Gannoruwa bat lyssavirus (GBLV)]. Experimental evidence suggests that the vaccines are not effective against phylogroup 2 (LBV, MOKV, SHIBV) or phylogroup 3 lyssaviruses (IKOV, West Caucasian bat lyssavirus, Lleida bat lyssavirus) (50, 52–56).

## Immune Response and Immune Evasion

Upon initial infection, the innate immune response is triggered in the periphery and evidence suggests that this response is partially effective against even the most pathogenic strains, with some viral particles being eliminated (57). However, further clearance is not achieved as pathogenic strains poorly stimulate and inhibit the activation and maturation of dendritic cells, resulting in a poorer antibody immune response (58–60). This prevention of the maturation of DCs is achieved through the inhibition of the interferon (IFN) autocrine feedback loop that is dependent on JAK-STAT signaling, which is specifically inhibited by the P protein (61).

The ability of lyssaviruses to evade the immune response is directly correlated to its pathogenicity, with pathogenic strains inducing a minimal response and successfully evading immune clearance (18). All the RABV proteins are multifunctional, with roles in viral entry, replication and spread, as well as in the sequestration of the immune system – either directly or indirectly (62). This ability is reliant solely on the immune-suppressive capabilities of viral proteins - primarily being the P, G and N proteins. The P protein is typically involved in sequestering the innate immune response by inhibiting the production of multiple antiviral products such as MxA, OAS1 and IFN-stimulated gene products (62). Furthermore, the P protein inhibits type I IFN responses and subsequent innate and adaptive immune responses through the inhibition of various IFN-related signaling pathways (63–67). The evasion of IFN responses in infected neurons is likely to be essential for the spread of RABV through the PNS, enabling the virus to reach the brainstem and eventually the salivary glands for spread to a new host (57). Similarly, the N is also predominantly involved in the sequestration of the innate response, primarily through the inhibition of RIG-I activation (68–70). Apoptosis in macrophages, T cells (including infiltrating T cells in the CNS) and microglia plays an important role in immune evasion and is stimulated by the G protein of pathogenic strains (71, 72), which appears to assist in the effective infiltration, replication and spread of the virus in the CNS (36, 73, 74).

## Discussion

While rabies has arguably been recognized for thousands of years, there remain many gaps in scientific knowledge of the disease and its causal agents. The rapid detection of 10 novel lyssaviruses in the past two decades raises multiple public health concerns, with their broader distribution and possible public health impact being yet unknown (13, 75). While information relating to many of the lyssavirus species remains poor, studies suggest that sustained spillover events from non-RABV lyssaviruses are likely to be rare, as almost all lyssaviruses – except for RABV and ABLV – are restricted to a single host species (76). However, many lyssavirus species have only a single, or few, isolates, including the novel GBLV which has a recent common ancestor with ABLV (56). In addition, host shifts in areas where RABV is endemic are likely to remain undetected due to poor surveillance (76). While host shift events remain rare, their impact can be devastating. North America alone is endemic for multiple terrestrial RABV variants, each being resultant of a host shift event (77). While host shift events may be geographically restricted, the potential for the translocation of the virus through human means remains a distinct possibility and risk (78–81). For example, the largest epizootic in recorded history resulted from the human-mediated translocation of a raccoon from the south-east of the United States to the north-eastern states (82). Further evidence suggests that raccoon rabies was enzootic at low levels for many years before its detection, natural spread, and subsequent human translocation (83). The raccoon RABV variant now accounts for nearly 75% of all terrestrial rabies cases in the USA and resulted in a significant increase in the number of human exposures in those areas where it is endemic (84). Thus, despite the rabies-related viruses not posing a significant health threat at present, continued efforts need to be made to ensure public health safety based on the limited knowledge and surveillance data available.

Despite the availability of an effective prophylactic treatment before the onset of symptoms, there remains no cure once rabies symptoms manifest. In addition, the majority of immunopathological knowledge available pertains to RABV, with limited studies being available for the rabies-related lyssaviruses. Therefore, there is a need for continued investigation into the mechanisms of infection, disease progression, host biology and a better understanding of bat immunology. Over and above, there is a dire need for improved global surveillance for all lyssaviruses. Given the significant public health threat posed by dog-mediated RABV, such surveillance data should play a critical role in the elimination of the disease from those dog populations where it is still rampant due to a failure to effectively break transmission through mass vaccination.

## Author Contributions

TS: Conception, preparation of first draft, editing and final review. LN: Conception, editing and final review. All authors contributed to the article and approved the submitted version.

## Conflict of Interest

The authors declare that the research was conducted in the absence of any commercial or financial relationships that could be construed as a potential conflict of interest.

## Publisher’s Note

All claims expressed in this article are solely those of the authors and do not necessarily represent those of their affiliated organizations, or those of the publisher, the editors and the reviewers. Any product that may be evaluated in this article, or claim that may be made by its manufacturer, is not guaranteed or endorsed by the publisher.
